# Technology use as a sleep-onset aid: are adolescents using apps to distract themselves from negative thoughts?

**DOI:** 10.1093/sleepadvances/zpac047

**Published:** 2022-12-22

**Authors:** Alexandra Daniels, Meg Pillion, Benita Rullo, Jessica Mikulcic, Hannah Whittall, Kate Bartel, Michal Kahn, Michael Gradisar, Serena V Bauducco

**Affiliations:** College of Education, Psychology & Social Work, Flinders University, Adelaide, Australia; College of Education, Psychology & Social Work, Flinders University, Adelaide, Australia; College of Education, Psychology & Social Work, Flinders University, Adelaide, Australia; College of Education, Psychology & Social Work, Flinders University, Adelaide, Australia; College of Education, Psychology & Social Work, Flinders University, Adelaide, Australia; WINK Sleep, Adelaide, Australia; School of Psychological Sciences, Tel Aviv University, Tel Aviv, Israel; WINK Sleep, Adelaide, Australia; Sleep Cycle, Gothenburg, Sweden; College of Education, Psychology & Social Work, Flinders University, Adelaide, Australia; School of Behavioural, Social and Legal sciences, Örebro University, Örebro, Sweden

**Keywords:** sleep aid, teenagers, sleep disturbances, smartphones, sleep onset

## Abstract

**Study Objectives:**

The aim of this study was to; (1) explore whether adolescents use technology as distraction from negative thoughts before sleep, (2) assess whether adolescents who perceive having a sleep problem use technology as distraction more compared to adolescents without sleep complaints, and (3) collect qualitative information about which devices and apps adolescents use as a distraction.

**Methods:**

This study used a mixed-methods cross-sectional design, where 684 adolescents (*M* = 15.1, *SD* = 1.2, 46% female) answered both quantitative and qualitative questions about their sleep (perceived sleep problem, sleep onset time (SOT), and sleep onset latency [SOL]) and technology use as distraction from negative thoughts.

**Results:**

The majority of adolescents answered “yes” or “sometimes” using technology as a distraction from negative thoughts (23.6% and 38.4%). Adolescents who answered “yes” to using technology as distraction were more likely to report having a sleep problem, longer SOL, and later SOT, compared to adolescents who answered “no”. The most popular device to distract was the phone, because of its availability, and the most common apps used for distraction included YouTube, Snapchat, and music apps.

**Conclusions:**

This study shows that many adolescents use technology to distract themselves from negative thoughts, which may help them manage the sleep-onset process. Thus, distraction may be one mechanism explaining how sleep affects technology use, rather than vice versa.

**Statement of Significance**

The scientific and popular literature have been heavily focused on technology use as a *cause* of adolescents’ sleep problems. However, many adolescents view technology as both a sleep stealer and a sleep *aid*. In this study, we asked adolescents whether they used technology in the evening as distraction from negative thoughts. The results showed that many do, especially adolescents who reported sleep difficulties. We also asked adolescents which devices/apps they prefer as distractive and why. This qualitative information suggested that adolescents both seek distraction from negative thoughts (e.g. watching videos) and actively regulate negative emotions (e.g. contacting friends). Theoretical models and recommendations to the public need to consider that technology may help adolescents to manage negative thoughts at bedtime.

## Introduction

Theoretical models for the links between evening technology use and sleep have been proposed and developed for more than a decade [1–4]. These models suggest that technology exerted its effects on sleep via different mechanisms, including (1) alertness induced by bright screens, (2) physiological and cognitive arousal from stimulating technological activities, and (3) the displacement of bedtime due to technology delaying the onset of sleep. To date, the collective evidence only supports the latter mechanism (i.e. bedtime displacement [5, 6]). However, there is scattered evidence throughout the scientific literature for a completely different connection between pre-sleep technology use and sleep. One link that has not been included in the original, nor subsequent, theoretical models [3, 7] is considering the other direction of this bi-directional relationship—whether sleep difficulties may influence technology use.

In our original illustrative model [1] we inserted arrows moving from technology use to sleep, implying that the direction of effect was that using technology close to bedtime *caused* sleep problems. However, there have been cross-sectional [8, 9], qualitative [10], and longitudinal studies [11–13] that suggest the opposite direction of effect—that sleep problems may influence evening technology use. For example, a longitudinal study assessing technology use and sleep problems in college students failed to find an association between increases in technology use and subsequent sleep problems [11]. Instead, the researchers discovered the opposite—that an increase in sleep problems was followed by increases in technology use [11]. This direction of effect has been confirmed in another longitudinal study of young adolescents, along with the finding that technology use also influenced adolescents’ sleep 1 year later [12]. These bi-directional effects were replicated in a recent longitudinal study of young adolescents [13]. Thus, there is longitudinal evidence from early adolescence to young adulthood that sleep problems can affect technology use.

Whilst the mechanisms by which sleep may affect evening technology use are not yet clear, here we propose that technology use may serve one understudied purpose—cognitive distraction. Cognitive distraction is a technique used in cognitive therapies for insomnia to manage negative thoughts and emotions [14]. There is research spanning 16 years that provides clues that adolescents may use technology in the evening for such purposes. In their cross-sectional study, Eggermont and van den Bulck surveyed 2546 adolescents about whether they used various technological devices as sleep aids [8]. About 60% used music as a sleep aid, 37% used TV as a sleep aid, and up to 28% of adolescents reported using video games as a sleep aid [8]. Similar findings have also been discovered for adults (mean age = 46 years [9];). Whilst it was clear that technological devices were commonly endorsed as a sleep aid by adolescents and adults, it remains unknown *why* people choose this coping mechanism.

Adolescents possess a later chronotype as they develop [15], which increases the likelihood of an extended sleep onset latency (SOL) if attempting to sleep at a socially desirable time (i.e. to be ready for school the next morning). Many adolescents report negative pre-sleep cognitions whilst attempting sleep [16–19], which aligns with ecological momentary research showing that the evening is a peak time for ruminative self-focus [20, 21]. Over time, adolescents may learn to distract themselves from pre-sleep negative thoughts and emotions by engaging in technology use [11]. Indeed, preliminary evidence shows young adolescents who have difficulty falling asleep escape negative feelings by engaging with social media [22]. Although these authors found adolescents used evening technology to escape negative emotions, it appeared this may be a coping mechanism that does not improve their sleep difficulties [22].

To the best of our knowledge, there remains limited evidence that has investigated if adolescents might be using technology to distract themselves from negative thoughts—and whether it is a helpful strategy to aid the sleep onset process. Furthermore, it appears that less is known about which specific devices and apps adolescents might use today for distraction. Consequently, the first aim of the present study was to explore whether adolescents use pre-sleep technology to distract themselves from negative thoughts. The second aim of the present study was to assess whether adolescents using technology to distract themselves from negative thoughts have similar rates of sleep problems compared to their peers who do not use technology as a distraction. The third aim of the study was to gain a better understanding of adolescents’ reasons and preferences for using specific devices and apps as a distraction in the evenings, thus utilizing a mixed-method approach.

## Method

### Participants and procedure

Six hundred and eighty-four adolescents aged between 12 and 18 years (*M* = 15.1, *SD* = 1.2, 46% female) were recruited from eight high schools of medium to high socioeconomic status, in the Adelaide metropolitan area between June and September 2019. The present study used opt-out consent, whereby the parents/guardians needed to return a form if they did not want their adolescent involved, or if they wanted to withdraw their adolescent’s data from the study. Nineteen participants did not provide their consent to partake in the research and were excluded from the study, 14 participants/survey entries were removed due to incomplete answers, and 20 were removed due to nonsensical responses. Thus, 631 cases were included in analyses.

The survey was delivered via the online program Qualtrics. Consenting participants were given the link to the survey during class time. Adolescent participants accessed the survey through a web link on their laptops, tablets, or computers. Participants’ data were stored anonymously to ensure participant confidentiality and anonymity with only relevant researchers having access to the data.

The present study was part of a larger study investigating technology use and sleep [23] and took an average of 30 min to complete. At the completion of the survey, all participating schools received AUD$100 and were offered a presentation from an expert in sleep psychology for the participating students. Ethics approval for this research was granted by the Flinders University Social and Behavioral Research Ethics Committee, the Department of Education and Child Development (DECD) and the Catholic School’s Board.

### Design

This study used a mixed-method cross-sectional design. The survey included both quantitative and qualitative questions to gain a deeper understanding of adolescents’ use of technologies as a sleep aid (i.e. which devices/apps and why they were chosen).

### Measures

#### Benefits of evening technology use.

Several questions were designed for this study to assess whether technologies were used in the hour before bed as a distraction from negative and distressing thoughts, and if so, what those technologies and applications were, and why they were used. Questions included; “Do you use any form of technology to help distract yourself from negative or distressing thoughts in the hour before bed?” with possible responses being “yes,” “no,” or “sometimes.” If an affirmative response (i.e. yes or sometimes) was indicated, participants were asked to “Please select the one technology you would most likely use to distract yourself from any negative or distressing thoughts”. A response was selected from a multiple-choice list of technologies (i.e. phone, iPad, laptop, desktop computer, iPod/mp3 player, television, gaming console, or “other” to allow participants to select a technology not listed by researchers). This question was then followed up by the question “Why would you choose that particular type of technology to help you over other forms of technology”, where participants were offered a free text response to answer. These questions were then repeated for apps (i.e. “Please select the one app you would most likely use to distract yourself from any negative or distressing thoughts”). A response was selected from a list of applications (i.e. messaging, phone calls, Instagram, Facebook, Snapchat, Twitter, YouTube, Reddit, Tumblr, Spotify/iTunes/Apple Music, Netflix/Stan, Viber/WhatsApp, gaming app, audiobook, or “other” to ensure other apps could be provided). Again, a free text response was then allowed to answer the question “Why would you choose that app to use over any other app?”. These additional free-response questions aimed to qualitatively assess adolescents’ perspectives on their evening technology use as a distraction, a strategy that sought to improve the quality and validity of the answers.

Furthermore, to determine whether or not using a technology was beneficial for aiding sleep, two questions were used. The first question determined helpfulness by asking “On a scale of 1 (not at all helpful) to 5 (extremely helpful), how helpful is this technology in distracting you from those negative or distressing thoughts?”. The following question assessed difficulty by asking “On a scale of 1 (not at all difficult) to 5 (extremely difficult), how difficult would you find it to fall asleep without using this technology?”.

#### Sleep problems.

Participants’ perception of whether or not they had a sleep problem was also assessed [24]. Participants were asked “Do you think that you have a sleep problem?” with the possible answers being “yes” or “no”. If “yes” was selected participants were presented with the open-ended question: “Why do you think you have a sleep problem?”.

#### Sleep variables

Adolescents completed a modified version of the School Sleep Habits Survey for adolescents [25] to determine: (1) the time at which participants attempted to sleep, “On school nights, what time do you turn the lights off and attempt to sleep?”, measured in clock time, and (2) SOL, “How long does it usually take you to fall asleep on school nights?”, measured in hours (either, 0, 1, or 2) and minutes (5-min increments, e.g. 10, 15 min). Attempted sleep time and SOL were then added together to determine the sleep variable of sleep onset time (SOT) which indicated the typical time at which the adolescent fell asleep.

#### Demographics

Participants were asked their gender (male, female, and prefer not to say), age, year level, postcode (to calculate SES), and the school they attended.

### Statistical analysis

All analyses were performed in SPSS v 26. A Chi-square test was used to compare the frequency of technology use as a distraction from distressing thoughts in adolescents with versus without a sleep problem. One-way ANOVAs were used to compare SOT and SOL in adolescents using versus not using technology as a distraction. Finally, we used thematic analyses to analyze the qualitative data and explore which devices and applications were the most popular distractions and why. We used a reflexive approach, where the first author (AD) developed codes and subsequent themes [26]. This process followed an inductive approach (i.e. guided by the content of the answers rather than preexisting ideas) and a semantic approach (i.e. reflecting the explicit content of the answers). The codes and themes were then discussed among the group to facilitate the interpretation of the data and finalize the themes.

## Results

Table 1 reports the descriptive statistics for each variable, including SOL, SOT, and, if adolescents reported they used technology as a distraction, how helpful they perceived technology as a sleep aid, and how difficult it would be to fall asleep without it.

**Table 1.** Means and standard deviations of the study variables

Variable (range)	*N*	*M*(*SD*)
SOL (0.00–175.00)	631	35.5 (37.2)
SOT (18.58–5.92)	616	23.18 (1.36)
Helpfulness (1–5)	382	3.7 (0.9)
Difficulty (1–5)	322	2.4 (1.1)

SOL, Sleep Onset Latency (in min); SOT, Sleep Onset Time in decimalized clock time, e.g. 23.5 = 23:30; Helpfulness = how helpful adolescents find technology in distracting them from negative or distressing thoughts (5 = extremely helpful); difficulty = how difficult adolescents would find it to fall asleep without using this technology (5 = extremely difficult).

### Prevalence of technology use as a distraction from negative or distressing thoughts

Sixty-two percent of adolescents responded “yes” or “sometimes” (23.6% and 38.4%, respectively) to using technology in the evening to distract themselves from negative or distressing thoughts. There was a significant difference in the proportion of adolescents with a sleep problem reporting to use of technology in the evenings as a distraction compared to those without a sleep problem, *χ*^2^ = 32.9*, p* < .001 (Table 2). Among adolescents who responded “yes” to using technology as a distraction, there was a larger percentage of adolescents with a sleep problem (36.8 vs. 18.3%). Among adolescents who responded “sometimes” to using technology in the evening to distract themselves, there was no statistically significant difference in proportions between those that had a sleep problem versus those who did not, *p* > .05. The proportion of adolescents who responded “no” to using technology as a distraction was significantly higher for those without a sleep problem, compared to those with a sleep problem (44 vs. 23%), *p* < .001.

**Table 2.** Cross-tabulations of adolescents with/without a sleep problem on the use of technology as a distraction (“yes”, “sometimes”, or “no”)

		Do you have a sleep problem?		Total
Do you use technology to distract yourself from negative or distressing thoughts?		No	Yes	
**Yes**	Count	82^a^	64^b^	146
	% Within sleep problem	18.3%	36.8%	23.5%
**Sometimes**	Count	169^a^	70^a^	239
	% Within sleep problem	37.7%	40.2%	38.4%
**No**	Count	197^a^	40^b^	237
	% Within sleep problem	44%	23%	38.1%

Each superscript letter denotes a subset of sleep problem categories whose column proportions (i.e. within each category of the dependent variable) do not differ significantly from each other at the *p* > .05 level.

### SOT and SOL in adolescents using technology as a distraction versus not

**Figure 1.**
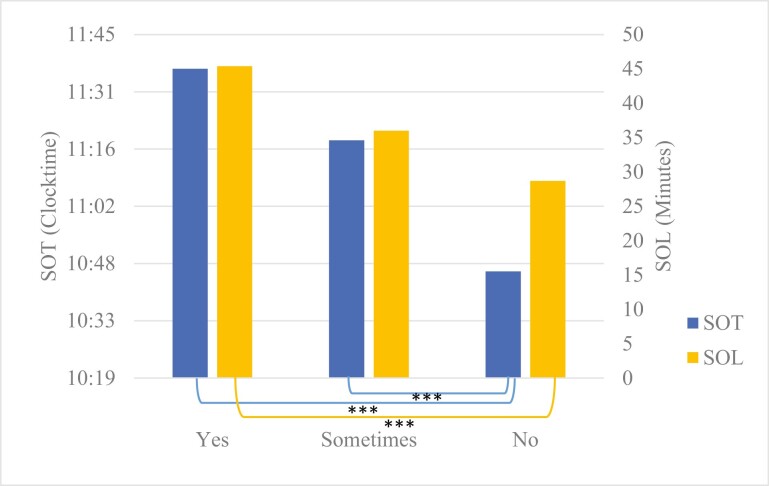
Sleep Onset Time and Sleep Onset Latency in adolescents who responded “yes”, “sometimes”, and “no” to the question “Do you use technology to distract yourself from negative or distressing thoughts in the hour before bed?”. SOT, Sleep Onset Time is in hours and minutes; SOL, Sleep Onset Latency is in minutes. ^a^The three asterisks indicate differences at *p* < .001.

SOT was significantly different among the different groups (“yes”, “sometimes”, and “no”—to using technology in the evening as a distraction), *F*(2, 623) = 20.6, *p* < .001, *η*^2^ = 0.06 (see Figure 1). SOT was not significantly later in the “yes” compared to the “sometimes” group (mean difference of 0.30, 95% CI [−0.21, 0.63], *p* = .07). However, SOT was significantly later in the “yes” group compared with the “no” group (mean difference of 0.84, 95% CI [0.52, 1.16], *p* < .001), a difference of 50 min. Finally, SOT in the “sometimes” group was significantly later compared with the “no” group (mean difference of 0.54, 95% CI [0.25, 0.82], *p* < .001), a difference of 32 min.

SOL was significantly different among the different groups, *F*(2, 623) = 9.57, *p* < .001, *η*^2^ = 0.03 (see Figure 1). SOL from the “yes” group was not significantly different compared with the “sometimes” group (mean difference of 9.3, 95% CI [−19.47, 0.81], *p* = .08). However, SOL was significantly longer in the “yes” group compared to the “no” group (mean difference of 16.7 min, 95% CI [−26.60, −6.83], *p* < .001). Finally, SOL from the “sometimes” group was not significantly longer compared to the “no” group (mean difference of 7.4 min, 95% CI [−14.93, 0.16], *p* = .06).

### Qualitative analyses

**Figure 2.**
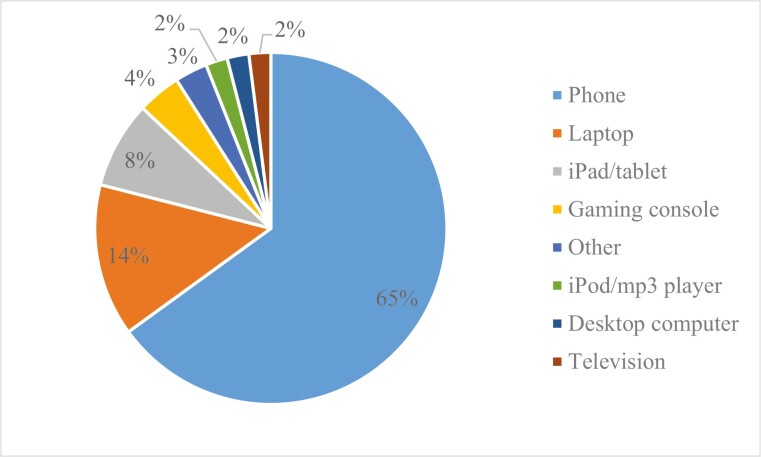
Percent distribution for the most to least popular technology.

**Figure 3.**
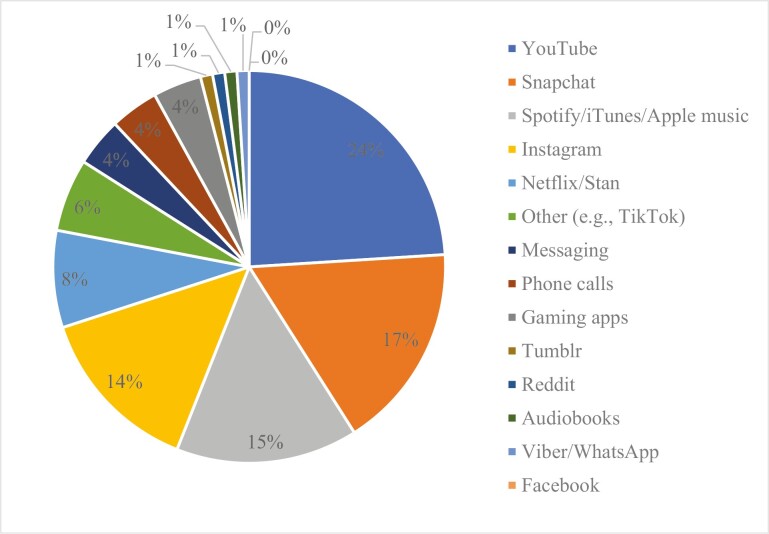
Percent distribution for the most to least popular application.

Adolescents were asked which technology—and subsequently which app—they would preferentially choose over any others to distract themselves from negative or distressing thoughts in the hour before bed. Figures 2 and 3 show the percent distribution for the most-to-least popular device and app, respectively. Thematic analysis of the reasons for each of these technology choices identified common themes. The phone was the most popular device because it has a diverse range of applications/uses (34%), is easily accessible/convenient (24.6%; e.g. “because my phone is always with me”), and it can be used to talk with friends (19.4%; e.g. “because it allows me to contact people that I want to talk to and it allows me to listen to music and watch videos of my favorite celebrities that keep me happy”). The most common two themes that emerged from using a laptop were that it was the favorite/only available technology (41.1%) and that it enables one to watch something such as a movie, show, or YouTube (37.3%; e.g. “because watching things on my laptop distracts me from everything else going on in my life”). iPad/tablets were chosen largely because they contained desired applications (58.8%), whereas gaming consoles were chosen predominantly due to their ability to improve mood or immerse oneself in a different world (58.8%; e.g. “I find video games to be the most immersive source of media. I can insert myself into the experience and it makes it easier to ignore what’s going on around me”).

When analyzing why the most popular apps were chosen, the following themes were identified. YouTube was used because it is entertaining (33%) and it is calming/takes one’s mind off oneself (30.9%; e.g. “I can watch entertaining videos that distract from any events occurring. Having sound from videos helps avoid any negative thoughts”). Further reasoning included, diverse content (18.1%), don’t know (9.6%), and favorite to use (8.5%). Snapchat was used to interact/communicate with friends (74.6%; e.g. “because it gives me the ability to contact my friends and ask for their advice or talk to them to get my mind off of it”), because it is the favorite app (13.6%), because it is fast/easy (6.8%), and unsure (5.1%). Spotify/iTunes/Apple music were used because listening to music is an enjoyable experience (42.6%) and is relaxing (35.2%; e.g. “because music keeps me happy when I am stressed, angry, upset or anxious about something. As I get to listen to my favorite artists that I love, there (sic) songs help me calm down”). Other themes included that the apps are distracting (16.7%) and the “don’t know” response (5.6%). One of the other most popular apps was Instagram, which was used because it is versatile and has lots of content (42.3%), “don’t know” (19.2%), it is useful to pass the time (13.5%), it is their most used app (13.5%) and it is used to message friends (11.5%; e.g. “because you can message on there, search for videos and also just scroll through your feed and the stories on there”).

## Discussion

The aim of this study was to explore an understudied mechanism in the link between technology and sleep in adolescents. Namely, whether adolescents use technology to distract themselves from negative thoughts which may *facilitate* sleep.

We found that more than half (62%) of the adolescents in our sample used, or sometimes used, technology as a distraction from negative and unpleasant thoughts in the evening. This was endorsed more among adolescents who reported having a sleep problem, which suggests that the association between evening technology use and sleep is more complex than first theorized (i.e. that evening technology use causes sleep problems). In practice, this also suggests that simply removing all technology from the bedroom might not be the best solution. Leaving many adolescents without this method to cope with their negative thoughts, might lead to more serious emotional health issues, such as sleep disorders and depression [27, 28]. Adolescents are prone to a longer SOL, due to the delay in their circadian rhythm, which creates an opportunity for worry and rumination before sleep [27]. Several studies both in adult [20, 21] and adolescent samples [18, 19] have shown that worry and rumination peak in the pre-sleep period. In turn, repetitive negative thinking is a transdiagnostic risk factor for the development and maintenance of depression [29]. Therefore, future studies into the links between technology use and adolescent sleep would do well to examine the role of negative thinking and depression in longitudinal and experimental studies. For example, an experiment could be designed to temporally measure negative thinking, sleep, and depression symptoms on two separate occasions - one night without technology use and one-night using technology as a distraction. Until more longitudinal and experimental studies are performed, we cannot exclude the possibility that negative thinking, sleep-onset difficulties, and technology use simply co-occur.

Adolescents who used technology as a distraction attempted their sleep later and took longer to fall asleep compared to adolescents who did not. This might mean that technology use does not help adolescents fall asleep faster [22]. Instead, the majority of adolescents report using technology to cope with the process of falling asleep. Previous qualitative studies have described adolescents’ struggle while waiting to fall asleep, where many feel the need to fill the time and avoid the negative thoughts that otherwise come to mind [30]. Some adolescents have depicted this process of waiting for sleep and distracting themselves as a vicious cycle, where one might pick up the phone again after attempting to sleep [30], a habit that is difficult to control [10]. Pre-bedtime technology use seems to be a double-edged sword—adolescents both identify it as a dependency and competing with their sleep but also as an integral part of their wind-down routine [10]. Whether adolescents would fall asleep quicker and sooner without devices remains an open question that can be answered using experimental and ecological momentary assessment designs. For example, one recent laboratory study with young adults found that 30 min of social media use (i.e. WhatsApp and Snapchat) before bed was not associated with later bedtimes compared to a neutral night, but progressive muscle relaxation was consistently associated with shorter SOL compared to the other conditions [31], suggesting that, perhaps, those who used technology as a distraction would still fall asleep at a similar time if only asked to refrain from using their phone.

One of the strengths of the present study was its mixed-methods design, which allowed us to garner more information from a large number of adolescents about their endorsement of using technology as a distraction—and why. Adolescents preferred to use their phones due to their versatility, which suggests that efforts to promote sleep health broadly in this population may wish to focus on this device. As to the content, the most used apps for distraction included YouTube, Snapchat, music apps, and Instagram. The reasons for using these apps varied from passive entertainment (e.g. YouTube and listening to music) to interacting with peers (e.g. Snapchat and Instagram), which is in line with a recent review [10]. Despite its passive use, YouTube was found to be a significant predictor of insufficient sleep (i.e. less than 7 hr of sleep [23]), likely because of its unstructured nature [32], whereas listening to music has shown a weaker association with sleep onset and duration [33] and is a common sleep aid (e.g. [8, 30]). Adolescents might engage in online social interactions well into the night that are motivated by fear of missing out (FoMO) (i.e. fear to miss important events, interactions, or activities when offline) [10]. Yet, online socializing is also an integral part of today’s peer interactions and friendships [34], and adolescents in this study expressed the need to connect with others in the evening to ease their worries. The qualitative information suggests that adolescents use technology to regulate their emotions, not only as a distraction, but also in other ways, such as seeking emotional support from friends. Future studies should investigate other emotion regulation strategies that are facilitated by technology use in the evening and how this in turn impacts adolescents’ sleep.

The main limitation of this study is its cross-sectional design, which does not allow conclusions regarding the true direction of the effect between technology use and sleep. Yet, our key questions clearly indicate the use of technology as a distraction from negative thoughts. The free text answers in the present study further supported the idea that adolescents use apps to distract themselves from unwanted thoughts in the evening. Future studies are needed to further explore this complex association. Experimental and ecological momentary studies would be particularly helpful to understand whether evening technology use does not only distract from negative thoughts but also from the designated bedtime (i.e. the proposed mechanism of time displacement), or even fuels negative thoughts (i.e. arousal mechanism), thus leading to sleep loss. Another limitation is that, due to the fast-paced development of new applications, the apps listed in this study need updating (e.g. TikTok became popular right after the data were collected). However, the theoretical implications of this study are not affected by the specific content of adolescents’ social media use. Finally, adolescents’ sleep onset might be a long and fragmented process where they re-engage with technology after finally attempting sleep. This fragmented process of sleep displacement [35] cannot be easily captured by sleep diaries/questionnaires but may be better detected by sleep-tracking devices.

## Conclusion

This study adds to the accumulating evidence of the complex relationship between sleep and technology use, showing that adolescents who are with sleep problems are more likely to use devices before bed. Specifically, adolescents expressed that technology distracts them from negative or unwanted thoughts, and previous research suggests social media may distract from negative emotions. Theoretical models as well as recommendations to adolescents, parents, and health professionals need updating to take into account that technology may be an integral part of many adolescents’ sleep routines, to help them regulate their negative thoughts.

## Data Availability

The data can be shared on reasonable request to the corresponding author.

## Notes

Note: This study was performed at Flinders University.
